# Effects of field-grown transgenic switchgrass carbon inputs on soil organic carbon cycling

**DOI:** 10.7717/peerj.7887

**Published:** 2019-10-16

**Authors:** Sutie Xu, Sarah L. Ottinger, Sean M. Schaeffer, Jennifer M. DeBruyn, C. Neal Stewart, Mitra Mazarei, Sindhu Jagadamma

**Affiliations:** 1 Department of Biosystems Engineering and Soil Science, University of Tennessee, Knoxville, TN, USA; 2 Department of Plant Sciences, University of Tennessee, Knoxville, TN, USA; 3 BioEnergy Science Center and Center for Bioenergy Innovations, Oak Ridge National Laboratory, Oak Ridge, TN, USA

**Keywords:** Transgenic switchgrass, Lignin downregulation, Soil organic carbon, Active carbon, Soil respiration, Soil quality

## Abstract

Genetic engineering has been used to decrease the lignin content and to change the lignin composition of switchgrass (*Panicum virgatum* L.) to decrease cell wall recalcitrance to enable more efficient cellulosic biofuel production. Previous greenhouse and field studies showed that downregulation of the gene encoding switchgrass caffeic acid *O*-methyltransferase (COMT) and overexpression of the switchgrass *PvMYB4* (MYB4) gene effectively improved ethanol yield. To understand potential environmental impacts of cultivating these transgenic bioenergy crops in the field, we quantified the effects of field cultivation of transgenic switchgrass on soil organic carbon (SOC) dynamics. Total and active SOC as well as soil respiration were measured in soils grown with two COMT-downregulated transgenic lines (COMT2 and COMT3), three MYB4-overexpressed transgenic lines (L1, L6, and L8), and their corresponding non-transgenic controls. No differences in total SOC, dissolved organic carbon (DOC), and permanganate oxidizable carbon (POXC) were detected between transgenic and non-transgenic treatments for both COMT (10.4–11.1 g kg^−1^ for SOC, 60.0–64.8 mg kg^−1^ for DOC, and 299–384 mg kg^−1^ for POXC) and MYB4 lines (6.89–8.21 g kg^−1^ for SOC, 56.0–61.1 mg kg^−1^ for DOC, and 177–199 mg kg^−1^ for POXC). Soil CO_2_-carbon (CO_2_-C) production from the COMT2 transgenic line was not significantly different from its non-transgenic control. In contrast, the COMT3 transgenic line had greater soil CO_2_-C production than its non-transgenic control (210 vs. 165 µg g^−1^) after 72 days of laboratory incubation. Combining the improvement in ethanol yield and biomass production reported in previous studies with negligible change in SOC and soil respiration, COMT2 could be a better biofuel feedstock than COMT3 for environmental conservation and cost-effective biofuel production. On the other hand, MYB4 transgenic line L8 produced more biomass and total ethanol per hectare while it released more CO_2_-C than the control (253 vs. 207 µg g^−1^). Long-term in situ monitoring of transgenic switchgrass systems using a suite of soil and environmental variables is needed to determine the sustainability of growing genetically modified bioenergy crops.

## Introduction

Using biofuels to meet our increased energy demand is a promising option which reduces consumption of petroleum-based fuels. Switchgrass (*Panicum virgatum* L.), a native warm season C_4_ perennial grass, is a popular bioenergy crop in North America because it produces high biomass and energy with increased nutrient and water use efficiency, and is adapted to grow in diverse environmental conditions. It is widely reported that using ethanol fuels in automobiles benefit the environment by releasing less greenhouse gases compared to gasoline (Spatari, Zhang & MacLean, 2005; Schmer et al., 2008). Despite environmental benefits from switchgrass-derived ethanol, the cell wall recalcitrance of switchgrass due to the presence of lignin poses challenges in using this crop as an efficient biofuel feedstock (Himmel et al., 2007). This is because cost-ineffective pre-treatments are required to break the lignin-polysaccharide matrix for the production of fermentable sugars (Fu et al., 2011b; Baxter et al., 2014). To improve the cost-effectiveness and efficiency in the conversion of lignocellulosic biomass to biofuel, switchgrass can be genetically engineered to change the amount and composition of lignin. Transgenic switchgrass derived by the downregulation of a lignin biosynthetic enzyme, caffeic acid *O*-methyltransferase (COMT), was shown to have higher sugar release and ethanol yield due to reduced lignin content and syringyl:guaiacyl (S/G) lignin monomer ratio, yet maintain similar biomass yield as non-transgenic switchgrass plants (Fu et al., 2011a; Baxter et al., 2014, 2016). Another transgenic switchgrass line with overexpressed enzyme *PvMYB4* (MYB4), a transcriptional repressor of many lignin biosynthetic genes, also had decreased lignin content, along with increased sugar release and biomass yield (Baxter et al., 2015).

As a biofuel crop, switchgrass can improve soil organic carbon (SOC) sequestration compared to other common bioenergy crops such as corn (McLaughlin & Walsh, 1998; Gauder et al., 2016). Because switchgrass is perennial, there is less soil disturbance, longer rooting depth, more allocation of below-ground biomass, higher root turnover and exudation, and better control of soil erosion in production systems. Thus, monitoring SOC changes can improve our understanding of the environmental impacts of transgenic switchgrass production. Lignin is a complex carbon (C) compound that is relatively recalcitrant to microbial decomposition and it may surround cellulose, hemicellulose, and protein in plant cell walls, limiting the accessibility of enzymes to these relatively labile C compounds (Potter & Klooster, 1997; Berg, Johansson & Meentemeyer, 2000; Pauly & Keegstra, 2008; Austin & Ballaré, 2010). For example, lignin had a negative effect on litter decomposition when *Arabidopsis thaliana* was manipulated to have two different lignin levels (Talbot & Treseder, 2012). Thus, it is conceivable that field grown transgenic plants with reduced lignin may speed up residue decomposition and negatively affect SOC accumulation and ecosystem sustainability. However, it is unclear if the reduced lignin content of transgenic switchgrass can influence SOC cycling when majority of the aboveground biomass is removed for biofuel production. In addition, the interaction of residue quality and SOC dynamics also depends on environmental conditions such as temperature and moisture. For example, the warm and humid climatic conditions prevalent in the southeastern US could exacerbate the residue quality-driven SOC loss via increased decomposition rates.

A study conducted by DeBruyn et al. (2017) reported that COMT-downregulated transgenic switchgrass did not change total SOC compared to non-transgenic control. However, the post hoc power analysis showed that chances of detecting true differences in SOC was low, suggesting that SOC change under transgenic switchgrass may be possible. Since detectable changes in total SOC may occur slowly, active SOC fractions such as dissolved organic carbon (DOC) and permanganate oxidizable carbon (POXC) can be considered as indicators for total SOC change (Silveira, 2005; Mandal et al., 2011; Culman et al., 2012). In addition, soil CO_2_ evolution can reveal differences in microbial activity due to biochemical alteration of plant inputs which affect the formation and stability of total SOC (McLauchlan & Hobbie, 2004).

Our objective was to determine the changes in total and active SOC and microbial activity in COMT-downregulated and MYB4-overexpressed transgenic switchgrass field production systems. We collected soils from two on-going COMT and MYB4 transgenic switchgrass field experiments to understand the changes in total SOC, DOC, POXC, and CO_2_-carbon (CO_2_-C) production between transgenic and non-transgenic lines. Our hypothesis was that transgenic switchgrass plant inputs containing lower lignin content will increase active fractions of SOC and microbial activity compared to non-transgenic switchgrass plant inputs.

## Materials and Methods

### Study site and experimental design

This study was conducted at the University of Tennessee East Tennessee Research and Education Center, Knoxville, TN (35.53°N, 83.57°W), leveraging two on-going transgenic switchgrass field experiments with COMT and MYB4 transgenic plants. The soil in the study site belongs to Shady series (fine-loamy, mixed, subactive, thermic Typic Hapludults). The COMT experiment was established in 2011 in a completely randomized design (CRD) with ten transgenic replicates and five corresponding non-transgenic replicates for each transgenic line. The plots were separated from each other by 160 cm to avoid interference. Each plot had nine vegetatively propagated clones which were planted 80 cm apart. To reduce shading effects, wild-type switchgrass plants of the “Alamo2” genotype were used as border plants. The detailed information on this field experiment was described in Baxter et al. (2014, 2016) and DeBruyn et al. (2017). For the COMT study, two independent T1-generation events (COMT2 and COMT3) produced by RNAi-mediated gene silencing of COMT (Fu et al., 2011a) were compared with their corresponding non-transgenic controls.

The MYB4 experiment was established in 2012 with three replicates for each of the transgenic lines and controls that are arranged in CRD. Each plot had four vegetatively propagated T0 clones planted 76 cm apart and separated by 152 cm from each other. “Alamo” plants were grown as border around the field site. The experimental details are included in Baxter et al. (2015). For this study, four independent transgenic events (L1, L2, L6, and L8) generated from overexpressing MYB4 in an Alamo-derived switchgrass parental clone ST1 (Shen et al., 2013) and three independent non-transgenic ST1 controls derived from tissue culture were considered. Since L2 was identified as chimeric for the transgene (Baxter et al., 2015), it was excluded from this study.

### Soil sampling and soil organic carbon analysis

Soil samples were collected in September 2016 from 15 to 20 random locations around the switchgrass clumps in each plot at a depth of 0–10 cm and homogenized into one composite sample per plot. Each soil sample was mixed thoroughly and passed through a 2 mm sieve. A sub-sample was stored at 4 °C for incubation experiments and the rest was air-dried for total and active SOC analysis. Air-dried soils, further pulverized by a mortar and pestle, were used for the determination of total SOC by dry-combustion method using a CN analyzer (Elementar vario TOC cube, Langenselbold, Germany). To measure POXC, the method described by Weil et al. (2003) and Culman et al. (2012) was used. Briefly, 18 ml deionized water was added to 2.5 g soils followed by the addition of 2 ml 0.2M potassium permanganate solution. The mixture was shaken for 2 min and incubated for 10 min in the dark at room temperature. After incubation, the extracts were diluted 100-fold and tested using a spectrophotometer (Thermo Scientific Evolution 60, Waltham, MA, USA). To measure DOC, 25 ml water was added into 5 g soils, shaken for 1 h, filtered using Whatman No. 42 filter paper, and the C concentration in the extract was determined using a TOC analyzer (Shimadzu TOC-VCPH, Kyoto, Japan).

### CO_2_ measurement

A 72-day laboratory incubation was conducted to determine CO_2_ respiration from the soils. For COMT, soils from both transgenic COMT2 and COMT3 as well as their non-transgenic controls were incubated. Seven replicate incubations were set up for each transgenic line and three for non-transgenic replicates. For MYB4, soils from transgenic events with medium to high level of MYB4 expression such as L8 and L1 with seven- and 16-fold *PvMYB4* expression levels, respectively, were incubated along with the non-transgenic control. Selection of these two lines was also based on the fact that they had the most positive (L8) and negative (L1) influence on biomass yield. The incubation experiment was conducted by transferring 25 g of fresh soil stored at 4 °C for 2–3 days into 500 ml glass jars. The moisture content of the soil was adjusted to 20% by adding the required amount of water, depending on the moisture content of the collected soil, and mixed thoroughly. The jars were closed tightly and kept at room temperature in the dark. The CO_2_ respiration was measured on days 1, 3, 8, 15, 29, and 72. At each measurement time, 0.5 ml gas was taken from the headspace of the jars using a polypropylene syringe through the rubber septa on the jar lids, and injected into a CO_2_ Analyzer (LI-820; Li-Cor Inc., Lincoln, Nebraska, USA) to measure the CO_2_ concentration. To maintain the aerobic environment, jars were vented occasionally for 10 min using a hand-held fan.

### Statistical analysis

Data analysis was conducted using SAS version 9.4 (SAS Institute, 2014). The PROC TTEST procedure was used to test the difference in total SOC, POXC, and DOC between transgenic and non-transgenic treatments of COMT2 and COMT3. The PROC GLIMMIX procedure was used to compare different treatments from the MYB4 experiment. The three controls in the MYB4 experiment were not significantly different to each other so their mean value was compared with transgenic treatments. Differences were considered significant when *P*-values < 0.05. Fisher’s least significant difference method was used to compare mean differences. The CO_2_-C evolution from the transgenic COMT and MYB4 were compared with the corresponding non-transgenic controls by repeated measures ANOVA using the PROC GLIMMIX procedure.

## Results

In the COMT experiment, total SOC, DOC, and POXC of transgenic COMT2 and COMT3 were not significantly different from their non-transgenic controls. Mean values across all the COMT treatments ranged from 10.4 to 11.1 g kg^−1^ for total SOC, 60.0 to 64.8 mg kg^−1^ for DOC, and 299 to 384 mg kg^−1^ for POXC (Table 1). Similarly, all three transgenic lines of MYB4 had statistically similar total SOC, DOC, and POXC to the non-transgenic lines. Mean values across all MYB4 treatments ranged from 6.9 to 8.2 g kg^−1^ for total SOC, 56.0 to 61.1 mg kg^−1^ for DOC, and 177 to 199 mg kg^−1^ for POXC (Table 1).

**Table 1:** Soil organic carbon, dissolved organic carbon, and permanganate oxidizable carbon of transgenic and non-transgenic switchgrass lines. Each data point indicates average of measured parameters.

Treatment	SOC (g kg^−1^)	DOC (mg kg^−1^)	POXC (mg kg^−1^)
COMT2			
Non-transgenic	10.4	64.8	299
Transgenic	11.1	63.3	313
*P*	0.299	0.705	0.735
COMT3			
Non-transgenic	10.7	60.0	384
Transgenic	10.9	63.6	322
*P*	0.742	0.309	0.350
MYB4			
Non-transgenic	7.11	57.5	190
Transgenic L1	6.89	56.0	198
Transgenic L6	8.21	59.8	199
Transgenic L8	7.40	61.1	177
*P*	0.254	0.767	0.992

**Figure 1:**
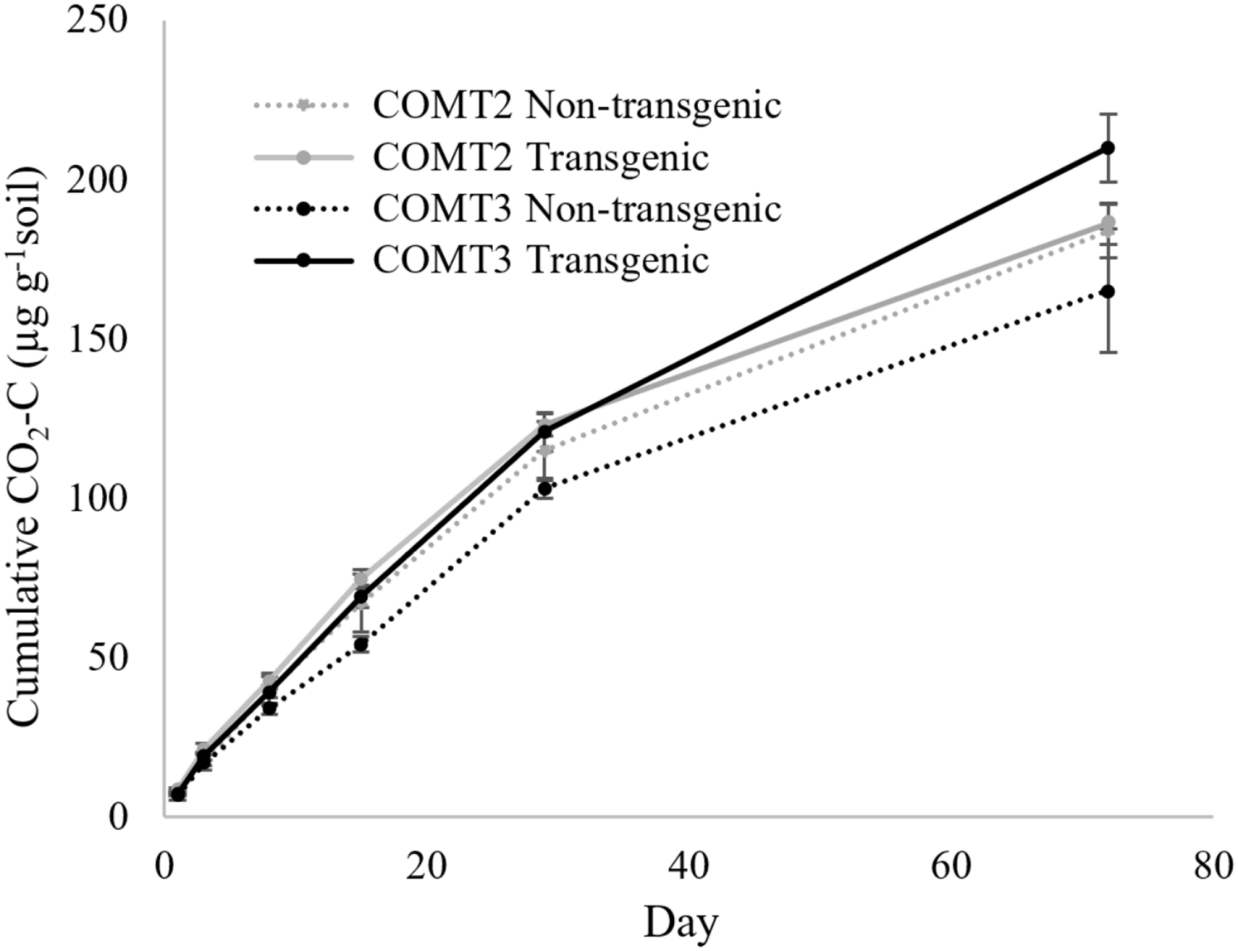
Cumulative CO_2_ production from transgenic and non-transgenic COMT lines. Each data point indicates cumulative CO_2_ production until the day of incubation.

The COMT2 transgenic and non-transgenic lines had statistically similar soil CO_2_ efflux throughout the incubation period (Table 2) with cumulative CO_2_-C production at the end of incubation (day 72) of 186 µg g^−1^ soil for COMT2 transgenic line and 184 µg g^−1^ soil for the non-transgenic line (Fig. 1). In contrast, a treatment × time interaction was significant for COMT3 (*P* = 0.011) (Table 2). On day 72, the COMT3 transgenic line had 27% higher cumulative CO_2_-C than its corresponding non-transgenic line (210 vs. 165 µg g^−1^ soil) and the difference was significant only after 1 month of incubation (Fig. 1). When the cumulative CO_2_-C from the two transgenic COMT lines were compared, a significant treatment × time interaction was found (*P* < 0.0001). The difference between the two lines was not detected in the first month of incubation, while the cumulative CO_2_-C from transgenic COMT3 was 13% higher than that from COMT2 on day 72.

**Table 2:** Analysis of variance corresponding to the comparison of the CO_2_ respiration across treatments. Each data point indicates ANOVA *P*-values.

Effect	COMT			MYB4		
	C2 vs. C3	C2 vs. Con	C3 vs. Con	L1 vs. L8	L1 vs. Con	L8 vs. Con
	*P*					
Treatment	0.814	0.433	0.204	0.021	0.423	0.048
Time	<0.0001	<0.0001	<0.0001	<0.0001	<0.0001	<0.0001
Treatment × time	<0.0001	0.864	0.011	0.0006	0.251	0.096

**Figure 2:**
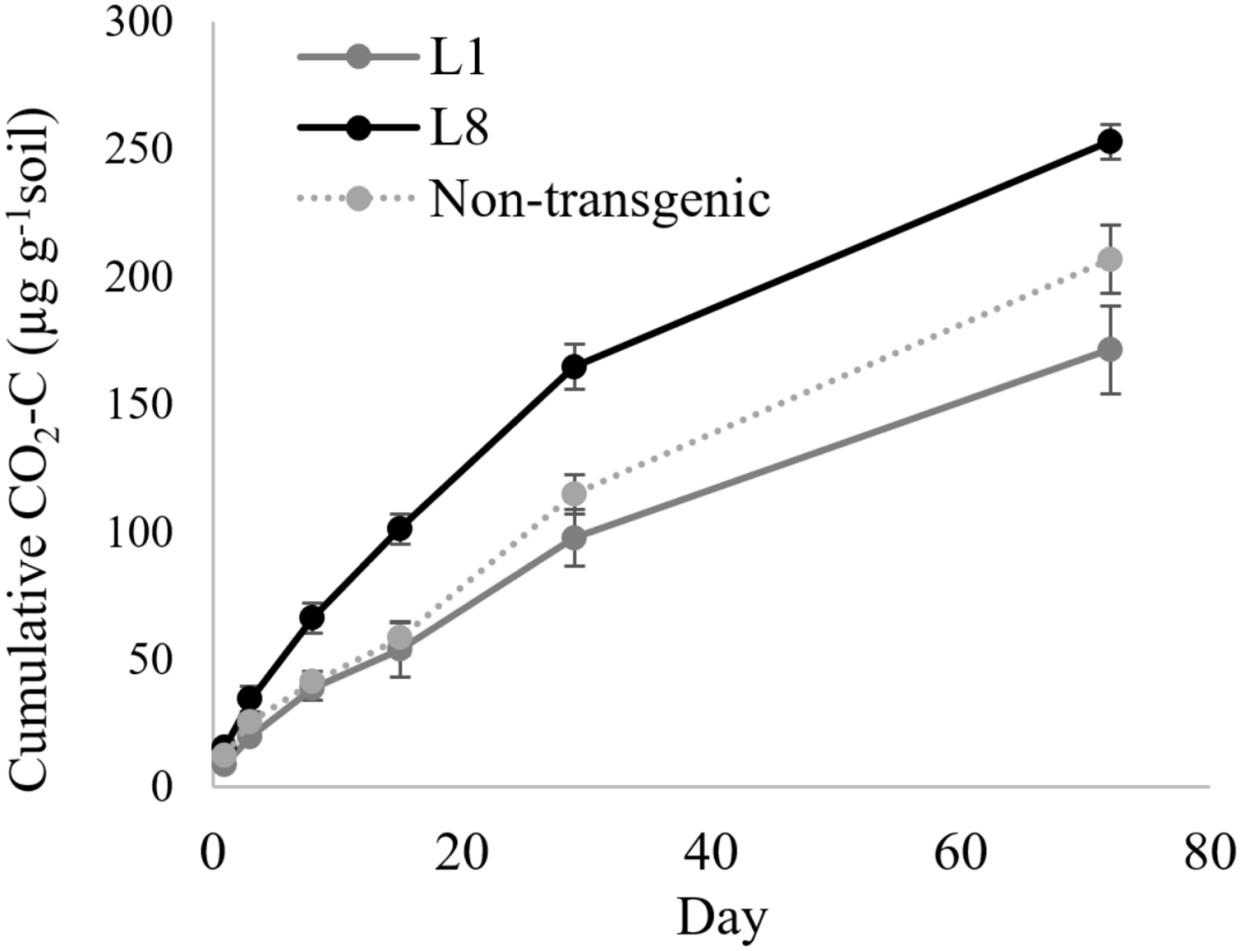
Cumulative CO_2_ production from transgenic and non-transgenic MYB4 lines. Each data point indicates cumulative CO_2_ production until the day of incubation.

In the case of MYB4 lines, the treatment × time interaction was significant when the cumulative CO_2_-C was compared between MYB4 transgenic lines L1 and L8 (*P* = 0.0006, Table 2); L8 produced more CO_2_-C compared to L1 after 2 weeks of incubation (Fig. 2). The cumulative CO_2_-C was 87%, 68%, and 48% higher in L8 than L1 on day 15 (101 vs. 54 µg g^−1^ soil), day 29 (165 vs. 98 µg g^−1^ soil), and day 72 (253 vs. 171 µg g^−1^ soil), respectively. The soil CO_2_-C production of L1 was statistically similar to that of the non-transgenic control during the 72-day incubation, but L8 had higher cumulative CO_2_-C than the control throughout the incubation period (*P* = 0.048).

## Discussion

Transgenic switchgrass has increasingly become a viable cellulosic biofuel feedstock, but lignin downregulation, while improving the biofuel conversion efficiency, could be a concern from the standpoint of SOC sequestration in soils where the plants are grown. This study was an extension of DeBruyn et al. (2017) which found that total SOC at the beginning of growing season (June) of COMT lines after 5 years of field establishment was not significantly different between transgenic and control lines. Here, we (i) sampled soils in September after 6 years of establishment, so plant roots had more time to interact with the soils, (ii) measured active as well as total SOC pools, and (iii) included one more transgenic switchgrass type, MYB4, which was not examined by DeBruyn et al. (2017). Since total SOC change occurs very slowly, we expected that active fractions of SOC (DOC and POXC) that are more sensitive to ecosystem management would show difference between transgenic and non-transgenic lines. However, our results showed that both COMT and MYB4 transgenic lines with altered lignin content for 5–6 years under field conditions did not reduce active or total SOC compared to non-transgenic lines. Earlier studies showed that COMT downregulation and MYB4 overexpression decreased lignin content and S/G lignin monomer ratios in the aboveground biomass (Baxter et al., 2014, 2015, 2016). The lack of SOC response to the biochemical difference in aboveground biomass could be due to the fact that aboveground biomass was removed from the field by the end of each growing season, and therefore SOC was controlled by the belowground C input. DeBruyn et al. (2017) found no change in root lignin content due to COMT downregulation, suggesting that the biochemical differences seen in aboveground biomass are not present belowground, and thus not impacting soil SOC dynamics. No negative effect of transgenic switchgrass cultivation on total and active SOC suggest that transgenic switchgrass has the potential to improve cost-effectiveness and efficiency of biofuel production without compromising the quality of soil. This finding is consistent with the previous study showing that soil properties such as total SOC, pH, and concentrations of 19 soil elements (e.g., nitrogen, phosphorus) were not changed by COMT downregulation (DeBruyn et al., 2017). It should be noted that in production systems, the aboveground biomass may not always be removed, so future research should include evaluation of the SOC dynamics under different rates of aboveground biomass removal.

Transgenic crops have the potential to influence soil microbial activity by changing the root exudate production and microbial community composition (Motavalli et al., 2004). Our study measured soil respiration as a proxy for microbial activity and found that the effect of genetic modification on soil CO_2_-C production varied with different lines. For example, transgenic COMT3 produced more CO_2_-C than its non-transgenic counterpart, but that was not observed for COMT2. Baxter et al. (2014) found that COMT2 also outperformed COMT3 in terms of biomass yield and ethanol production. Nonetheless, bacterial diversity, richness, and community composition determined via 16S rRNA gene amplicon sequencing was not different between transgenic COMT and the non-transgenic lines (DeBruyn et al., 2017). Another study investigated microbial communities using metagenomics and metatranscriptomics, finding no differences in the major phyla of microbial community between transgenic and non-transgenic COMT lines at this site (Chauhan et al., 2014). Taken together, we conclude that COMT2 is a more promising option as a biofuel feedstock than COMT3 as it provides additional benefit in terms of less CO_2_ emission from soil to the environment.

The transgenic MYB4 line L1, which had the highest MYB4 expression level relative to the non-transgenic control (16-fold), produced a similar amount of CO_2_-C to the control. On the contrary, L8, with only sevenfold increase in MYB4 expression, produced more CO_2_-C compared to the control. Baxter et al. (2015) showed that, among the MYB4 transgenic lines, L1 had the highest reduction in lignin content (12%) and S/G lignin monomer ratio (12%), and the highest increase in sugar release (41%) and ethanol yield efficiencies (69%) compared to the control. Nevertheless, the plant morphology of L1 was changed dramatically by genetic modification resulting in decrease in biomass production by 79% and number of tillers by 17% (Baxter et al., 2015). On the other hand, the L8 transgenic line had increased biomass production by 63% compared to its non-transgenic control despite marginal decrease in lignin content (7%), no change in S/G lignin monomer ratio and sugar release efficiency, and only 28% increase in ethanol yield efficiency (Baxter et al., 2015). We suspect that the increased CO_2_-C production from L8 transgenic line could be due to increased below-ground biomass input concomitant with the higher aboveground biomass production. Since this study was conducted after only 5–6 years of planting transgenic switchgrass, long-term monitoring of the environmental impacts is warranted. Over the long-term, transgenic lines which can produce higher above- and below-ground biomass, such as L8, could potentially increase both the efficiency of biofuel production as well as SOC sequestration. We also speculate that the below-ground allocation of C inputs from transgenic switchgrass plants, especially at deeper soil depth, may be useful in rebuilding degraded soils if they can be grown successfully in nutrient and water-limited areas and/or on marginal lands.

## Conclusions

Our study revealed that COMT-downregulated and MYB4-overexpressed transgenic switchgrass accumulated the same amount of total and active SOC pools compared to their non-transgenic controls, suggesting that transgenic switchgrass systems sustain soil quality while improving the efficiency of biofuel production. We also found that some transgenic lines significantly increased soil respiration compared to their non-transgenic counterparts in controlled lab-scale experiments. Therefore, further in situ studies for the detailed understanding of how plant-root-microbial interaction changes with the cultivation of transgenic bioenergy crops is necessary. Our study was conducted after 5–6 years of cultivation and we collected soil samples only once and only from 0 to 10 cm soil depth for studying limited soil properties. Long-term monitoring of in-field emission of greenhouse gasses and multiple soil variables at lower soil layers across different experimental sites will be beneficial to determine the optimal gene modification of bioenergy crops to benefit both biofuel production and environmental conservation.

## Supplemental Information

10.7717/peerj.7887/supp-1 Supplemental Information 1 Soil organic carbon, dissolved organic carbon, and permanganate oxidizable carbon raw dataset. Each data point corresponds to measured value from the laboratory.

## Additional Information and Declarations

### Data Availability

The following information was supplied regarding data availability: The raw data from the laboratory measurements are available as a Supplemental File.
